# SOX9-regulated cell plasticity in colorectal metastasis is attenuated by rapamycin

**DOI:** 10.1038/srep32350

**Published:** 2016-08-30

**Authors:** Estefania Carrasco-Garcia, Lidia Lopez, Paula Aldaz, Sara Arevalo, Juncal Aldaregia, Larraitz Egaña, Luis Bujanda, Martin Cheung, Nicolas Sampron, Idoia Garcia, Ander Matheu

**Affiliations:** 1 Cellular Oncology group, Biodonostia Institute, San Sebastian, Spain; 2 Department of Gastroenterology, Hospital Donostia and Instituto Biodonostia, University of the Basque Country, Centro de Investigacion Biomedica en Red en Enfermedades Hepaticas y Digestivas (CIBERehd), San Sebastian, Spain; 3 School of Biomedical Sciences, Li Ka Shing Faculty of Medicine, The University of Hong Kong, Hong Kong, China; 4 IKERBASQUE, Basque Foundation, Bilbao, Spain

## Abstract

The cancer stem cell (CSC) hypothesis proposes a hierarchical organization of tumors, in which stem-like cells sustain tumors and drive metastasis. The molecular mechanisms underlying the acquisition of CSCs and metastatic traits are not well understood. SOX9 is a transcription factor linked to stem cell maintenance and commonly overexpressed in solid cancers including colorectal cancer. In this study, we show that SOX9 levels are higher in metastatic (SW620) than in primary colorectal cancer cells (SW480) derived from the same patient. This elevated expression correlated with enhanced self-renewal activity. By gain and loss-of-function studies in SW480 and SW620 cells respectively, we reveal that SOX9 levels modulate tumorsphere formation and self-renewal ability *in vitro* and tumor initiation *in vivo*. Moreover, SOX9 regulates migration and invasion and triggers the transition between epithelial and mesenchymal states. These activities are partially dependent on SOX9 post-transcriptional modifications. Importantly, treatment with rapamycin inhibits self-renewal and tumor growth in a SOX9-dependent manner. These results identify a functional role for SOX9 in regulating colorectal cancer cell plasticity and metastasis, and provide a strong rationale for a rapamycin-based therapeutic strategy.

Cancers display a high degree of heterogeneity between individual patients but also between cancer cells within the same tumor. Both types of heterogeneity affect clinical practice. During the last decade, it has been demonstrated that there is a population of cancer cells with stem-like properties, so-called cancer stem cells (CSCs), in several types of malignancies. CSCs are defined by their abilities to self-renew and generate differentiated progeny. These characteristics enable them to be the root of malignancies and to play a major role in tumor initiation and recurrence, therapy resistance and metastasis^1^.

Colorectal adenocarcinoma is the second most commonly diagnosed type of cancer and constitutes the second leading cause of cancer-related mortality worldwide, causing nearly 700,000 deaths per year^2^. Colorectal cancer occurs sporadically in the majority of cases, being due to inherited mutations in less than 10% of patients. In most patients, death is not caused by the primary tumor, but rather by its metastasis in other organs and associated complications. Indeed, patients are generally diagnosed at an advanced stage, wherein the 5-year survival rate is only 11.7%^2^.

Ten years ago, various research groups demonstrated the existence of colorectal cancer stem cells (CR-CSCs), and revealed these cells to be responsible for treatment resistance^3^. More recently, it has been identified that CR-CSCs have a role as drivers of the metastatic progression of colorectal cancer^4^. Moreover, the expression of the CD44v6 variant of CD44 or CD110 was shown to serve as a biomarker for this CR-CSC pool of colorectal metastasis drivers^5^,^6^. These studies started to unravel the mechanisms involved in the regulation of CR-CSCs associated with metastasis and showed that plasticity between CR-CSCs and non-CR-CSCs occurs at advanced stages of tumor progression.

The transcription factor Sex-determining region Y (SRY)-box 9 (SOX9) plays a crucial role in stem cell maintenance and lineage commitment during embryonic development and also in adult tissue homeostasis. In the intestinal epithelium, lineage tracing and loss of function mouse models identified that Sox9 is a key regulator of tissue homeostasis, regeneration and tumor initiation, through its functions in stem/progenitor cell maintenance and Paneth cell differentiation^7^,^8^,^9^,^10^,^11^. These activities arise acting as an effector and at the same time regulator of Wnt signaling^10^, a pathway whose activation is sufficient to initiate colorectal tumors, that is relevant for the maintenance of CR-CSCs^12^. Moreover, it is the most frequently aberrantly activated pathway in colorectal cancer^13^.

There is growing evidence of the impact of SOX9 in human malignancies^14^,^15^,^16^,^17^. In particular, several studies have revealed that SOX9 is commonly overexpressed in colorectal cancers^18^,^19^,^20^,^21^,^22^,^23^, even in cases where the gene is mutated, event which happens in around 5–10% of cases^13^,^24^. Clinico-pathologically, high SOX9 expression correlates with tumor progression and advanced tumor stage^18^ and has been associated with lower overall patient survival^20^,^21^. Functional studies have supported the view that SOX9 plays a pro-oncogenic role in primary colorectal cancer cells^18^,^21^, but under some circumstances it behaves as a tumor suppressor^25^,^26^. The role of SOX9 in the regulation of CR-CSCs has not been previously explored. In this work, we found that SOX9 is sufficient and necessary for the acquisition and maintenance of CR-CSC and metastatic traits, properties linked to transcriptional and post-transcriptional regulation. Finally, we reveal that SOX9-mediated self-renewal and growth is impaired by the mTOR inhibitor rapamycin.

## Results and Discussion

### High levels of SOX9 correlate in CR-CSCs and metastatic cells

**Figure 1:**
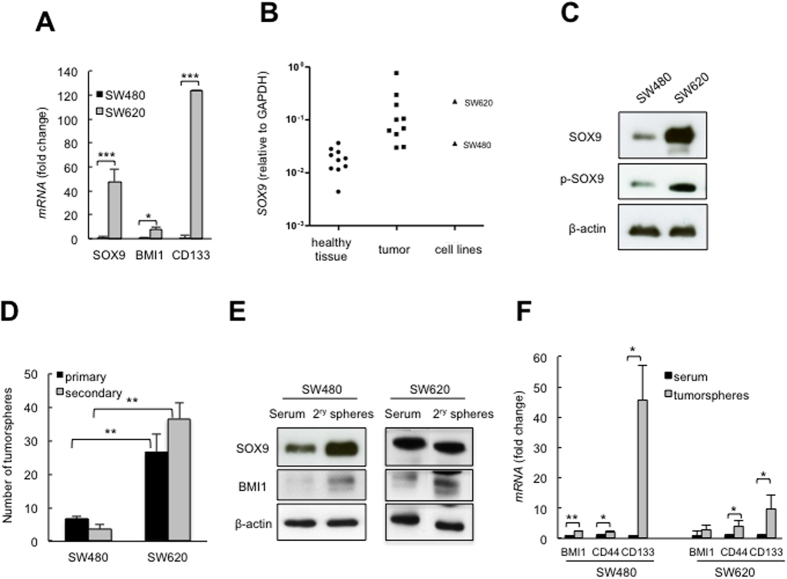
High levels of SOX9 correlate with CR-CSCs. (**A)** Higher expression of stem cell markers in SW620 than in SW480 cells (n ≥ 3). (**B)** *SOX9* mRNA expression relative to GAPDH in normal and tumoral colonic paired human samples and in SW480 and SW620 cell lines. (**C)** Higher SOX9 protein expression and phospho-SOX9 (S181) in SW620 cells than in SW480 cells. (**D**) Number of tumorspheres derived from SW480 and SW620 cells (n = 6). (**E)** SOX9 and BMI1 protein expression in tumorspheres and in parental SW480 and SW620 cells (n = 3). **(F)** Stem cell marker mRNA expression levels in tumorspheres relative to the corresponding parental cells (n ≥ 3).

SW480 and SW620 cell lines were derived from a primary colorectal adenocarcinoma and its lymph node metastasis, respectively^27^. We started by characterizing the expression of CR-CSC markers^3^, finding that metastatic SW620 cells had higher levels of *BMI1*, *CD133* and *SOX9* than SW480 primary cells (Fig. 1A). These differences were markedly strong in the case of the last two genes. When *SOX9* levels were compared with CRC human samples and matched adjacent colon tissue, we found *SOX9* expression significantly increased in CRC tissues (p < 0.05), with levels in SW620 being between the most highly expressed cancer tissues, whereas in SW480 were near the lowest cases (Fig. 1B). Therefore, the level of expression of SOX9 in SW480 and SW620 cell lines is within the range of overexpression observed in human colorectal samples, suggesting that their levels are of biological relevance. SOX9 protein levels were also strikingly elevated in SW620 cells as well as phosphorylated SOX9 at serine 181 (Fig. 1C). This site is known to stimulate SOX9 transcriptional and DNA-binding activity^28^, indicating that SOX9 upregulation in metastatic cells is associated with transcriptional and post-transcriptional modifications. In clinical samples, SOX9 expression is higher in liver metastasis than matched primary colorectal cancers, where it is part of an aggressive stem cell signature together with ASCL2, LGR5, EPHB3 and ETS2^29^. Hence, our molecular identification of SOX9 is consistent with the clinical data, and together they show that SOX9 exhibits a dynamic expression in colorectal cancer, with high levels of SOX9 being associated with CR-CSCs and metastasis.

Next, we observed that the metastatic cells exhibited greater capacity to form tumorspheres (primary) than SW480 cells (26.6 in SW620 *vs*. 6.7 in SW480). Moreover, the ability for self-renewal, measured in terms of the number of secondary tumorspheres, was also much higher in SW620 cells (Fig. 1D). In agreement with the enrichment in the CR-CSC pool, we detected elevated expression of *CD133*, *CD44* and BMI1, as well as SOX9, in tumorspheres formed from both cell lines (Fig. 1E,F). The above information, together with the evidence that both cell lines belong to the stem-like subtype showing high Wnt activity^30^, postulates SW480 and SW620 as suitable models to study the role of SOX9 in cellular plasticity and metastasis.

### SOX9 overexpression provides stemness properties to colorectal cancer cells

**Figure 2:**
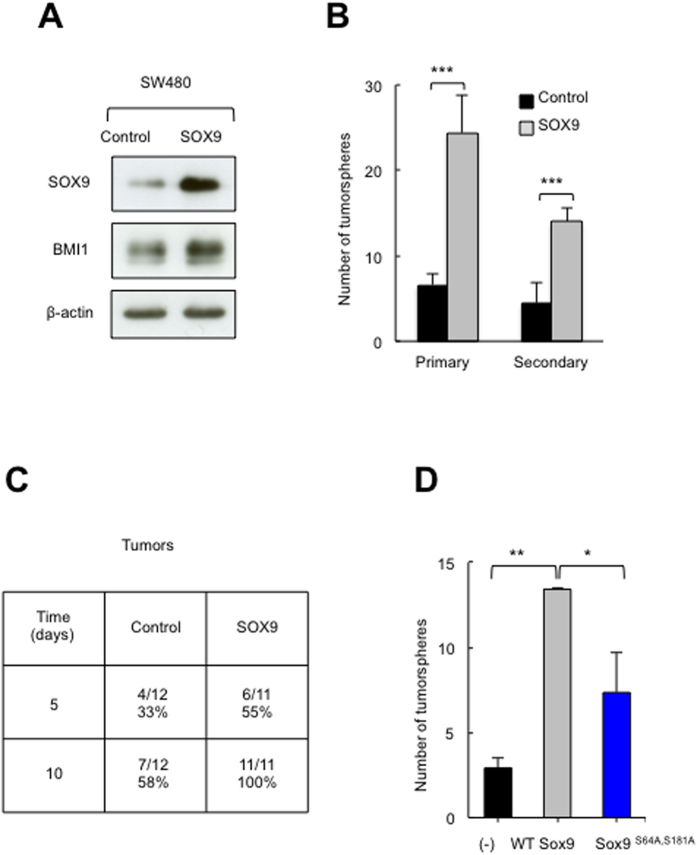
SOX9 overexpression provides self-renewal ability. (**A)** Representative immunoblots of SOX9 and BMI1 in SW480 cells transduced with SOX9 (SOX9) or empty vector (control) (n = 3). (**B)** Number of primary and secondary tumorspheres in control and SOX9 overexpressing SW480 cells (n ≥ 3). **(C**) Tumor initiation ability of indicated genotypes. Frequency of tumors formed in immunocompromised mice after subcutaneous injection of 1·10^6^ cells. (**D**) Number of tumorspheres formed in control (−), *Sox9*^S64A,S181A^ and WT Sox9 SW480 transduced cells (n = 3).

To determine whether SOX9 activity is involved in the plasticity between non-CR-CSCs and CR-CSCs, we used a lentiviral vector harboring a plasmid with the SOX9 coding sequence to produce SOX9-overexpressing SW480 cells, and compared their functional properties to control empty-vector transduced cells. Western blotting revealed the overexpression of SOX9 in SW480 cells (Fig. 2A). Importantly, the ability to form tumorspheres was markedly different between control and SOX9 overexpressing cells. Indeed, cells with SOX9 overexpression generated 4-fold greater number of primary tumorspheres and 2.5-fold higher secondary tumorsphere formation (Fig. 2B). Next, we determined the effect of SOX9 overexpression on tumor initiation, a distinctive feature of CSCs^1^. Notably, by 10 days, all immunodeficient Foxn1^nu^/Foxn1^nu^ mice inoculated with SOX9 overexpressing cells had developed tumors, compared to just over half (58%) of tumors derived from control cells (Fig. 2C). These results indicate that SOX9 promotes the acquisition of CR-CSC characteristics, both *in vitro* and also *in vivo*.

Given the differences observed in phosphorylated SOX9 between primary and metastatic cells, we wondered whether this activity might play a role in the gain of stemness. For this, we transduced SW480 cells with a construct containing point mutations at S64 and S181 phosphorylation sites (Sox9^S64A,S181A^) or WT-Sox9^31^ and compared them to non-infected cells. We found that Sox9^S64A,S181A^ cells formed fewer tumorspheres (0.65 fold) than WT-Sox9 and slightly more than controls (Fig. 2D). Another study has recently found that SLUG prevents SOX9 ubiquitin-mediated proteasomal degradation, thereby controlling its stability and maintaining lung CSC activity^32^. To our knowledge, these studies have provided the first proofs of a role for SOX9 post-translational modifications associated with cancer phenotypes.

**Figure 3:**
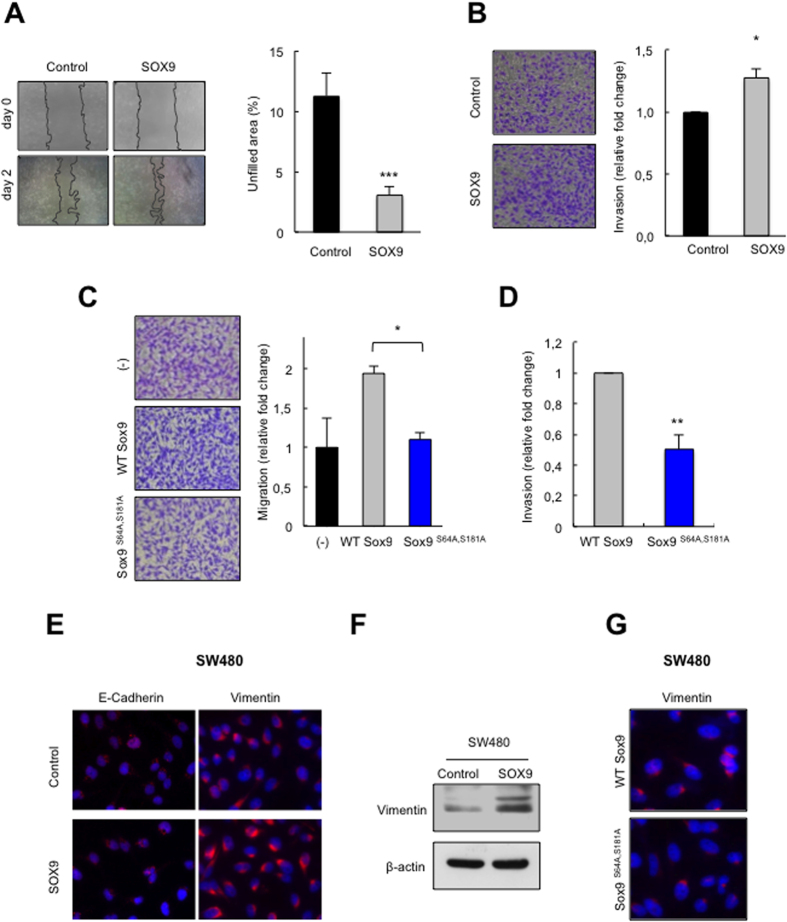
SOX9 contributes to gain of metastatic traits in CRC cells. (**A**) Representative image and quantification of non-filled area in wound healing assays of SW480 control and SOX9 overexpressing cells (n = 4). (**B)** Representative images and quantification of transwell invasion for the indicated genotypes (n ≥ 3). (**C)** Representative images and quantification of transwell migration of SW480 control (−) compared to cells transduced with *Sox9*^S64A,S181A^ or a WT form of SOX9 (WT-*Sox9*) (n = 2). (**D**) Quantification of transwell *in vitro* invasion for the indicated conditions (n = 2). (**E)** Representative images of immunofluorescence studies of E-Cadherin and Vimentin in control and SOX9 overexpressing SW480 cells (n ≥ 2). (**F)** Representative Western blot of Vimentin in the indicated genotypes (n = 2). (**G**) Vimentin expression detected by immunofluorescence in SW480 cells transduced with the wt (WT Sox9) or the *Sox9*^S64A,S181A^ mutant form of SOX9 (n = 2).

Since cancer cell motility and consequent invasion of the basement membrane have been associated with the gain of CSC properties and an epithelial mesenchymal transition (EMT) program^33^, we investigated the effect of ectopic SOX9 overexpression on these phenotypes. Stable overexpression of SOX9 resulted in a significant increase in the migratory potential of SW480 cells (Fig. 3A). Collagen invasion assays showed that high levels of SOX9 also enhanced their invasive potential (Fig. 3B). Moreover, migration ability (Fig. 3C) and invasive potential (Fig. 3D) were impaired in Sox9^S64A,S181A^ cells, indicating that SOX9 phosphorylation is necessary, at least in part, for these processes. Next, we measured the expression of several EMT markers and detected a reduction in the expression of the epithelial adhesion protein E-Cadherin (Fig. 3E). In addition, SOX9 overexpression provoked a robust induction of the mesenchymal marker Vimentin (Fig. 3E,F), which was reduced in cells lacking the phosphorylation sites (Fig. 3G). A previous study found that ectopic SOX9 induced an epithelial mesenchymal transition (EMT) and led to the formation of more metastasis *in vivo* in an additional primary colorectal cell line^21^. Hence, high levels of SOX9 confer motility, invasive properties and a mesenchymal phenotype to primary colorectal carcinoma cells, all of them important features for the translocation of a cancer cell from the primary tumor to a distant tissue in metastasis.

**Figure 4:**
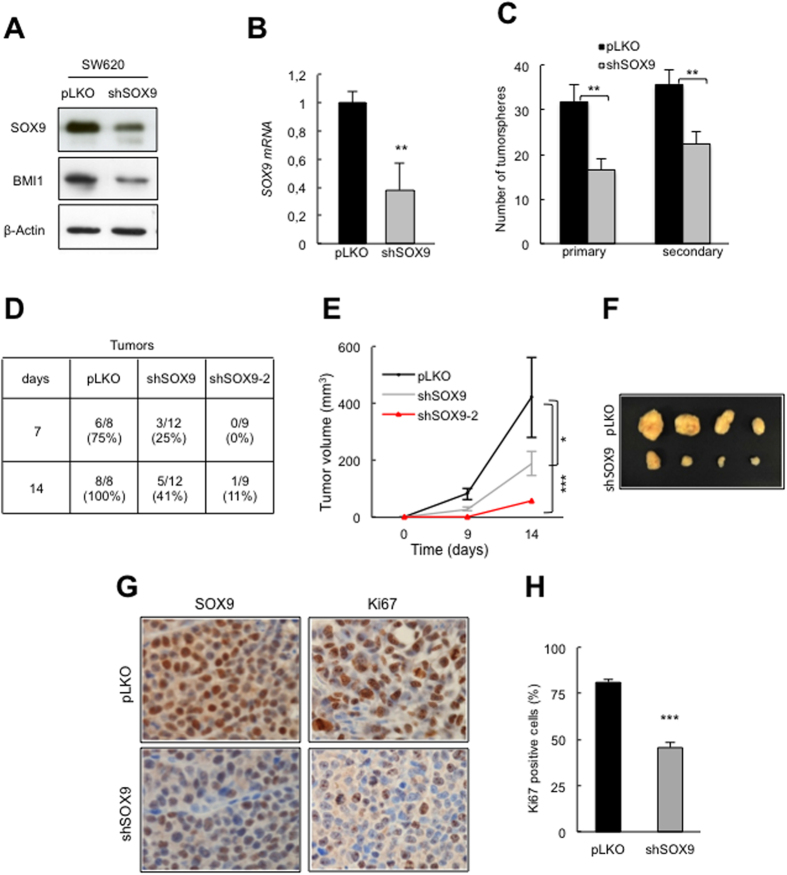
SOX9 silencing impairs self-renewal of SW620 cells. (**A)** Representative immunoblots of SOX9 and BMI1 protein expression in empty vector (*pLKO*) and *shSOX9* SW620 cells (n ≥ 3). (**B)** *SOX9* mRNA levels in *shSOX9* SW620 relative to *pLKO* cells (n = 3). (**C**) Plot of the number of primary and secondary tumorspheres in the indicated conditions (n ≥ 5). (**D)** Tumor initiation ability of the indicated cellular genotypes. Frequency of tumors formed in immunocompromised mice after subcutaneous injection of 1·10^6^ cells per injection. (**E**) Tumor volume calculated at the indicated time points in nude mice injected with *pLKO* or two independent *shSOX9* constructs (*shSOX9* and *shSOX9-2*) (1·10^6^ cells per injection). **(F)** Representative image showing the generated tumors per genotype. **(G)** Immunohistochemistry of SOX9 and Ki67 staining in SW620 derived tumors from pLKO or *shSOX9* conditions (n = 4). **(H)** Quantification of percentage of Ki67 positive cells in SW620 derived tumors from *pLKO* or *shSOX9* conditions (n = 4).

At a molecular level, we have previously described that Sox9 modulates proliferation directly regulating Bmi1^18^, a stem cell marker which genetic or pharmacologic inhibition irreversibly impairs CR-CSC activity^34^. Since we observed higher levels of both of them associated to CR-CSCs population (Fig. 1E), and in clinical samples^18^, we reasoned that BMI1 may be involved in SOX9-mediated colorectal cancer cell plasticity. In agreement with our hypothesis, the expression of BMI1 was higher in SOX9 overexpressing SW480 cells (Fig. 2A). In contrast, SW620 SOX9 silencing cells displayed BMI1 downregulation (Fig. 4A). ChIP-seq experiments revealed that SOX9 binds to the promoter of BMI1 in colorectal cells^35^, supporting that this regulation is direct. Overall, these data indicate that SOX9 modulates colorectal cancer cell plasticity regulating the dynamics of BMI1 and postulate SOX9-BMI1 as a critical axis for maintaining CR-CSCs activity and colorectal cancer pathobiology.

### SOX9 is necessary for CR-CSCs maintenance

Having demonstrated that SOX9 promotes the acquisition of self-renewal and metastatic traits in primary colorectal carcinoma cells, we next determined its function in metastatic cells. For this, we knocked down SOX9 in SW620 cells using a specific short hairpin RNA (*shSOX9*). Quantitative real-time PCR and Western blotting demonstrated the silencing of the endogenous expression of SOX9 by *shSOX9* (Fig. 4A,B). Strikingly, SOX9 downregulation resulted in a significant decline in the formation of both primary and secondary tumorspheres relative to that in SW620 control cells (Fig. 4C). Therefore, SOX9 silencing abrogates the self-renewal ability of colon metastatic cells *in vitro*.

To corroborate the functional need of SOX9 for CR-CSC maintenance, we moved onto *in vivo* experiments. The ability to initiate tumors was severely impaired in SOX9-silenced cells relative to control cells. Specifically, 41% of injections with *shSOX9* cells developed tumors, while tumors were generated in all mice injected with control cells (Fig. 4D). Similarly, only 11% of inoculations developed tumors when injected with an additional short hairpin targeting a different SOX9 sequence (*shSOX9-2*) (Fig. 4D). Furthermore, tumors originating from *shSOX9* and *shSOX9-2* cells grew more slowly than those from control cells (Fig. 4E,F). Strikingly, the impaired tumorigenic activity of SOX9 knockdown cells (Fig. 4G) was further corroborated *in vivo* by reduced cell proliferation in the tumors. *shSOX9* derived xenografts displayed lower number of Ki67 positive cells than tumors derived from control cells (p = 0.00049) (Fig. 4G,H). Together, these results show that the expression of SOX9 acts as a pleiotropic regulator maintaining self-renewal but also governing the proliferative capacity of colorectal cancer cells.

**Figure 5:**
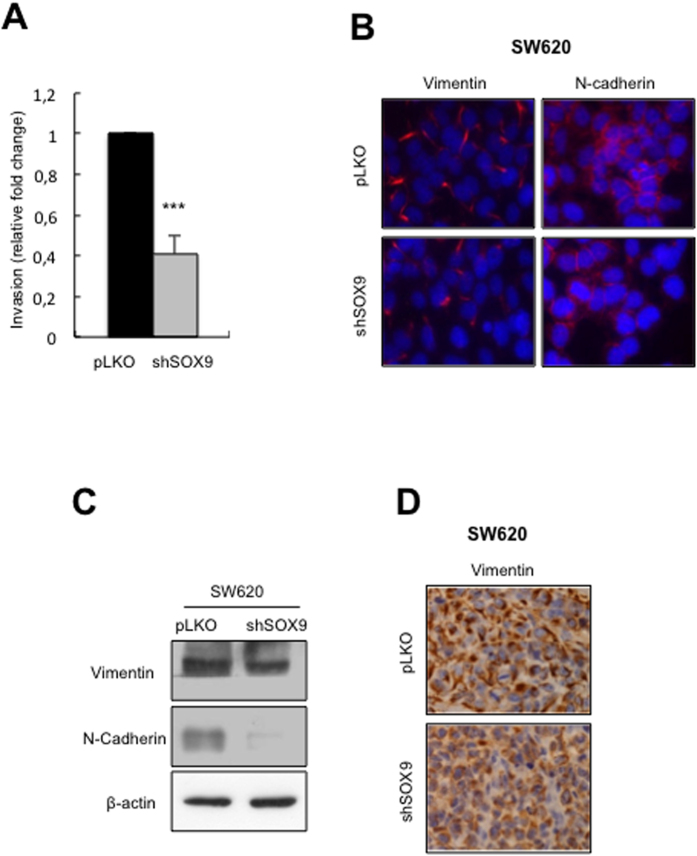
SOX9 silencing impairs metastatic phenotypes of SW620 cells. (**A)** Transwell invasion in SW620 *shSOX9* cells relative to SW620 control cells analyzed 24 hours after seeding (n = 3). (**B**) Vimentin and N-Cadherin mesenchymal markers expression analyzed by immunofluorescence in SW620 *pLKO* and *shSOX9* cells (n ≥ 2). (**C**) Representative western blot of Vimentin and N-Cadherin in SW620 *pLKO* and *shSOX9* cells (n ≥ 2). (**D)** Vimentin expression detected by immunohistochemistry in subcutaneous tumors generated by *pLKO* and *shSOX9* cells (n = 4).

Next, we studied whether SOX9-mediated loss of stemness properties could affect phenotypes necessary for metastatic colonization in distant organs^36^. Thus, the invasive potential of metastatic cells *in vitro* was impaired as a consequence of SOX9 silencing (Fig. 5A). Moreover, immunofluorescence and Western blot analysis revealed that the levels of the mesenchymal markers Vimentin and N-Cadherin were lower in SW620 cells in the absence of SOX9 *in vitro* (Fig. 5B,C). These results were further validated in tumors originated from *shSOX9* cells (Fig. 5D). In line with these findings, genome-wide chromatin immunoprecipitation with DNA sequencing ChIP-seq analysis identified genes involved in EMT and quiescence as targets of SOX9 in colorectal cancer cells^35^. In summary, our results demonstrate that SOX9 activity is required for retaining metastatic CR-CSC functional properties.

### SOX9 mediates rapamycin anti-tumorigenic effect

In CRC SOX9 overexpression is a strong predictor of shorter survival in 5-FU-treated patients and enhanced vascular invasion in biopsies^22^, whereas intestine stem cells expressing high levels of Sox9 are more resistant to irradiation in mice^37^. Since activation of CR-CSC signaling is central to acquired resistance to therapy in colorectal cancer, our results suggest that pharmacological inhibition of SOX9 might be a novel therapeutic approach for this type of cancer.

**Figure 6:**
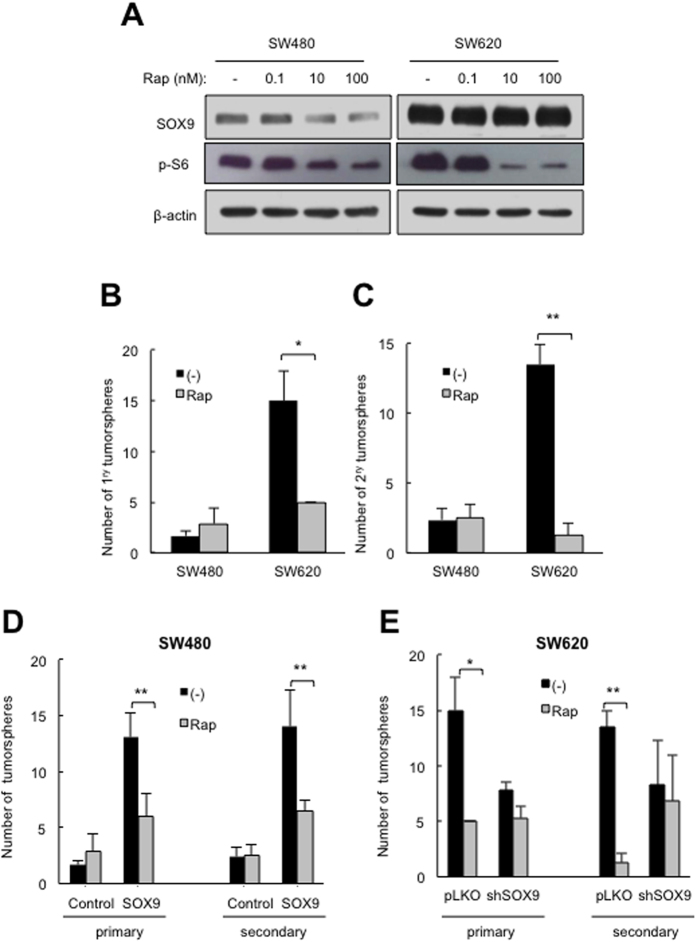
High SOX9 levels sensitize colorectal cancer cells to rapamycin. (**A)** Representative immunoblot of phospho-S6 Ribosomal protein (Ser235/Ser236) (p-S6), SOX9 and β-actin protein expression in SW480 and SW620 cells treated with the indicated concentrations of rapamycin for 24 hours (n = 2). (**B)** Number of primary tumorspheres in SW480 and SW620 cells treated with vehicle (−) or rapamycin 10 nM (Rap) (n = 4). (**C)** Number of secondary tumorspheres derived from SW480 and SW620 primary tumorspheres treated with vehicle (−) or rapamycin 10 nM (Rap) (n = 4). (**D**) Number of tumorspheres formed by control and SOX9 overexpressing SW480 cells in the presence or absence of rapamycin 10 nM (Rap) (n = 3). (**E)** Number of tumorspheres formed by *pLKO* and *shSOX9* SW620 cells in the presence or absence of rapamycin 10 nM (Rap) (n = 3).

It has been shown that rapamycin inhibits the early stages of colorectal tumorigenesis, concomitantly with decreasing Sox9 in the *Apc*^*fl/fl*^ mouse model^38^. Similarly, we have recently identified that rapamycin impairs glioma stem cell activity through silencing of SOX2 and SOX9 expression^39^. Given that the mammalian target of rapamycin (mTOR) is frequently activated in human colorectal cancers, and its natural inhibitor rapamycin and rapalogs are promising antitumor agents, whose efficacy is currently being tested in clinical trials with promising results^40^, we explored a potential relationship between the mTOR pathway and SOX9 in human colorectal cells. We observed that, in parallel to SOX9, the endogenous levels of phosphorylated S6 Ribosomal protein, downstream target and likely physiological effector of the mTOR pathway, were higher in SW620 than in SW480 cells (Fig. 6A). Next, we sought to establish whether rapamycin would be able to regulate metastatic cell activity through SOX9 activity. To answer this question, we first treated SW480 and SW620 cells with increasing concentrations of rapamycin (0.1, 10 and 100 nM) or vehicle (DMSO). SOX9 levels were not affected after cells cultured with the indicated concentrations of rapamycin (Fig. 6A). Given that the highest concentrations decreased the expression of SOX9 in glioma cells^39^, the effect of this agent on SOX9 expression seems to be context dependent. On the contrary, 10 and 100 nM concentrations of rapamycin decreased phosphorylated S6 more strongly in SW620 than in SW480 cells (Fig. 6A). This evidence supports the idea of enhanced impairment of mTOR signalling pathway in the metastatic cells. Moreover, we found that 10 nM of rapamycin was sufficient to cause a severe reduction in the formation of primary (66% decrease in rapamycin *vs*. vehicle treated) and secondary (90% reduction) tumorspheres in SW620 cells, without affecting that capacity in SW480 cells (Fig. 6B,C).

Next, we checked the response of CR-CSCs with modulated levels of SOX9 to rapamycin treatment. For this, we treated gain and loss of SOX9 expression SW480 and SW620 cells with rapamycin (10 nM) or vehicle and cultured them under CSC conditions. Strikingly, SOX9-induced tumorsphere formation and self-renewal in SW480 cells was markedly attenuated by the presence of a 10 nM dose of the mTOR inhibitor (Fig. 6D). In contrast, that low dose of rapamycin promoted a decrease of 66 and 90% in primary and secondary tumorspheres in SW620 control cells, whereas the decline was only of 32 and 18% respectively in *shSOX9* cells (Fig. 6E). These observations confirm that SOX9 levels sensitize CR-CSCs to rapamycin treatment, an effect likely mediated by the impairment of the mTOR signaling pathway, rather than the decline in SOX9 levels themselves.

**Figure 7:**
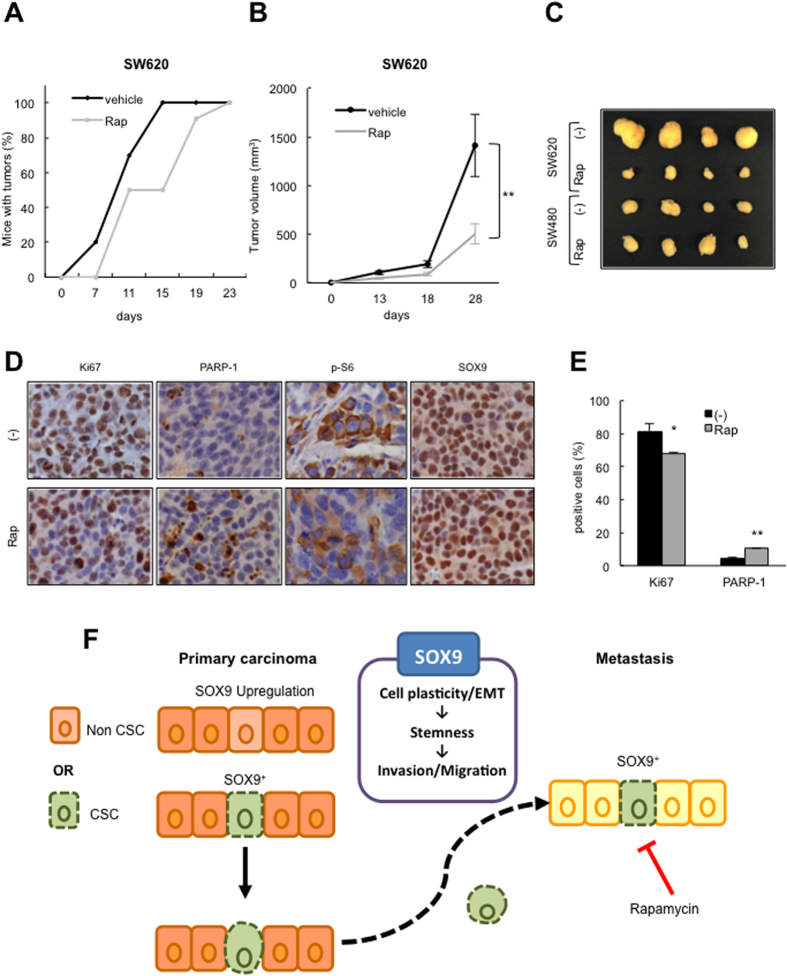
Rapamycin impairs tumor growth in SW620 metastatic cells. (**A)** Percentage of nude mice harbouring SW620 derived tumors at the indicated time points after intraperitoneal treatment with vehicle (−) or rapamycin 5 mg/Kg (Rap) twice a week (n = 8). Log-rank test (*p ≤ 0.05) (**B)** Representation of tumor volume formed by SW620 cells in immunocompromised mice treated with vehicle (−) or rapamycin 5 mg/Kg (Rap) (n = 8). (**C)** Representative image of tumors formed from SW620 and SW480 cells treated with vehicle (−) or rapamycin 5 mg/Kg (Rap). (**D)** Representative images of Ki67, fragmented PARP-1, p-S6, and SOX9 immunostaining in SW620 derived tumors in the indicated conditions (n = 4). (**E)** Quantification of percentage of Ki67 and fragmented PARP positive cells (n = 3). (**F)** Illustrative image of the role of SOX9 in CRC cell plasticity. The expression of SOX9 regulates stemness activity and EMT plasticity in CRC cells mediating the pro-metastatic abilities of migration and invasion. High SOX9 expressing cells exhibit enhanced CR-CSC properties subjacent to enhanced pro-metastatic abilities. The mTOR inhibitor rapamycin exerts an enhanced antitumoral and anti-metastatic effect on CRC cells expressing high SOX9 levels.

In order to validate the antitumor activity of rapamycin in cells with different SOX9 expression *in vivo*, we injected SW620 and SW480 cells subcutaneously in nude mice and treated animals with 5 mg/Kg of rapamycin twice a week. This treatment resulted in delayed tumor initiation and a significant decrease in tumor growth in SW620 cells (p < 0.01) compared to that observed with the vehicle control treatments (Fig. 7A–C). In contrast, the mTOR inhibitor did not affect tumor growth of SW480 cells (Fig. 7C). As shown in *in vitro* studies, phosphorylated S6 Ribosomal protein was markedly decreased whilst SOX9 showed similar expression in rapamycin compared to vehicle treated SW620 tumors (Fig. 7D). Finally, we also evaluated molecular markers for cell proliferation and apoptosis by immunohistochemistry. The expression of Ki67 was exclusively reduced (p < 0.05) in tumors obtained from SW620 cells treated with rapamycin (Fig. 7E and data not shown), whereas immunohistochemical staining for the apoptosis marker, fragmented PARP-1, was increased by rapamycin treatment (p = 0.004) (Fig. 7E). Together, these results show that rapamycin treatment attenuates CSC characteristics in colorectal cancer cells in a SOX9 expression dependent manner.

There is considerable focus nowadays studying the impact of cancer cell plasticity and metastasis. It is hint towards a complex network concerning heterogeneous pools of cells, which might interact within them or dynamically switch their characteristics^41^. These activities may be regulated in response to intracellular stress or microenvironmental stimuli via not well established molecular mechanisms yet. Our data indicate that the dynamics of SOX9 expression regulate colorectal cancer cell plasticity in a cell-autonomous manner. SOX9 directs cancer cell self-renewal and proliferation programs, governing the transition between CR-CSCs and non-CSCs. These functions are important for metastatic spread (Fig. 7F). Importantly, SOX9 inhibition impairs self-renewal and invasive potential, indicating that it might be considered as a novel therapeutic target for advanced colorectal cancers. In relation to this, we reveal that SOX9 levels define the antitumor action of the mTOR inhibitor rapamycin in colorectal cells, providing preclinical evidence to justify further research into therapeutic strategies based on this agent, using SOX9 levels as a biomarker for patient stratification.

## Materials and Methods

### Patients and tumor samples

Human colorectal carcinoma samples were provided by the Basque Biobank for Research-OEHUN (http://www.biobancovasco.org). The methods and experimental protocols in human samples were carried out in accordance with relevant guidelines, and all study participants signed informed consent form. The study was approved by the ethic committee of Biodonostia Institute and Hospital Donostia.

### Cell lines culture conditions

SW480 and SW620 cell lines were obtained from the ATCC (American Type Culture Collection) and cultured as adherent monolayers in DMEM medium (Invitrogen) supplemented with 10% fetal bovine serum. Tumorspheres were cultured in DMEM/F12 medium (Sigma) supplemented with 20 ng/mL of EGF and bFGF (Sigma) growth factors, in the presence of N2 and B27. For tumorspheres studies, 0.5·10^3^ cells/well were seeded in non-treated 12-well flat bottom plates and fresh medium was added every 3 days. After 10 days, primary (1^ry^) tumorspheres were counted. Then, spheres were disaggregated with Accutase, seeded for secondary (2^ry^) tumorspheres and maintained for another 10 days in culture.

### Viral infections

Lentiviral infections were performed as previously described^42^. For *gene* knockdown, cells were transduced with two independent constructs; shSOX9 (a gift from Dr. Bob Weinberg, Addgene plasmid #40644)^43^ and shSOX9-2 (Sigma TRCN0000342824). For SOX9 overexpression, we used the plasmid #36979 from Addgene, a gift from Bob Weinberg^43^. Plasmids with point mutations in S64 and S181 *(Sox9*^S64A,S181A^) or WT forms of Sox9 were gifts from Dr. Cheung. Cells were infected for 6 hours with a multiplicity of infection of 10.

### mRNA expression analysis

Total RNA was extracted with TRIzol (Life Technologies). Reverse transcription was performed using a High-Capacity cDNA Archive Kit (Life Technologies). Quantitative real-time PCR was performed using Power SYBR^®^ Green Master Mix (Thermo Scientific), in an ABI PRISM 7300 thermocycler (Applied Biosystems). Variations in RNA input were corrected using the expression of the GAPDH housekeeping gene. The ΔΔCT method was used for relative quantification.

### Western blot and immunofluorescence analysis

Immunoblot and immunofluorescence analysis were performed as previously described^18^. Primary antibodies used were: SOX9 (AB5535, Millipore), phospho-SOX9 (ab59252, abcam), BMI1 (05-637, Millipore), E-cadherin (BD610181, BD Transduction Laboratories), Vimentin (M7020, DAKO), N-Cadherin (BD610920, BD Transduction Laboratories), phospho-S6 Ribosomal protein (Cell Signaling Technology^®^, #4858) and β-actin (AC-15, Sigma). For Western blot detection of primary antibodies, we used HRP-linked antibodies (Santa Cruz Biotechnology) and detection was performed by chemiluminescence using NOVEX ECL Chemi Substrate (ThermoFisher). For immunofluorescence, secondary antibodies conjugated with fluorochromes were used and nuclear DNA was stained with Hoechst 33342 (Sigma). Images were obtained at a 40x magnification.

### Migration and invasion assays

For wound healing (*scratch*) assays, cells were seeded at a confluence of 90% in 24-well flat-bottom plates and 12 hours later a linear artificial gap (scratch) was made in serum-deprived conditions for 48 hours. The non-filled area (i.e., not covered by cell migration) was quantified using Scion image software (Scion Corporation). Transwell cell migration was evaluated using 6.5-mm Transwell^®^ chambers with 8.0-μm pore polycarbonate membrane inserts (Corning #3422). Invasion assays were performed using the QCM™ Collagen Cell Invasion Assay (ECM551, Millipore). Invading cells were quantified 24 hours after the seeding.

### *In vivo* carcinogenesis assays

For subcutaneous injection, SW480 and SW620 cells were harvested with trypsin/EDTA and resuspended in PBS. Cells (1·10^6^) were injected subcutaneously into both flanks of Foxn1^nu^/Foxn1^nu^ nude mice (8 weeks old). Mice were examined twice a week and external calipers were used to measure tumor size at the indicated time points from which tumor volume was calculated according to the formula ½(*length* × *width*^2^).

For rapamycin experiments, SW620 and SW480 cells were treated for 48 h with rapamycin 10 nM before subcutaneous implantation (1·10^6^ cells in PBS). Five days later, mice were injected intraperitoneally with rapamycin (5 mg/kg) or vehicle twice a week during the experiment. Tumor volume was estimated as described above.

### Immunohistochemistry

Tumors generated in mice were dissected, fixed in 10% formalin for 48h and embedded in paraffin. 4 micrometer-thick sections were incubated with primary antibodies (SOX9, AB5535 (Millipore); Ki67, ab15580 (Abcam); Vimentin, M7020 (DAKO); phospho-S6 Ribosomal protein (Cell Signaling Technology^®^, #4858), and fragmented PARP-1, 32064 (Abcam) at 37 °C for 2 hours. The sections then were washed and incubated with MACH 3 Rabbit/Mouse Probe and MACH 3 HRP-Polymer (M3R531, Biocare Medical). Immunostaining was developed with 3,3′Diaminobenzidine (DAB, SPR-DAB-060, Spring Bioscience).

### Data analysis

Data are presented as mean values ± S.E.M. with the number of experiments (n) in parenthesis. Unless otherwise indicated, statistical significance (p-values) was calculated using the Student’s t test. Asterisks (*, **, and ***) indicate statistical significance (p < 0.05, p < 0.01, and p < 0.001, respectively).

## Additional Information

**How to cite this article**: Carrasco-Garcia, E. *et al*. SOX9-regulated cell plasticity in colorectal metastasis is attenuated by rapamycin. *Sci. Rep*. **6**, 32350; doi: 10.1038/srep32350 (2016).
